# Uncovering a novel binding trench in ERRα: insights from molecular simulations

**DOI:** 10.3389/fmolb.2025.1523932

**Published:** 2025-02-27

**Authors:** Lamees Hegazy

**Affiliations:** ^1^ Center for Clinical Pharmacology, Washington University School of Medicine in Saint Louis and University of Health Sciences and Pharmacy in Saint Louis, St. Louis, MO, United States; ^2^ Department of Pharmaceutical and Administrative Sciences, Saint Louis College of Pharmacy, University of Health Sciences & Pharmacy in St. Louis, St. Louis, MO, United States

**Keywords:** molecular dynamics simulations, ligand recognition, estrogen-related receptor, drug discovery, novel binding trench, dynamic ligand binding

## Abstract

Although estrogen-related receptor α (ERRα) holds significant therapeutic potential for treating various disorders, developing selective agonists remains challenging due to the poor pharmacokinetics and limited selectivity of current ligands. This study presents unconstrained molecular dynamics simulations of ERRα bound to an agonist ligand, uncovering dynamic ligand-binding behavior as the ligand shifts between two orientations: one in the orthosteric pocket and another in a newly identified trench adjacent to this site. The free energy landscape reveals that both binding orientations are comparably populated, with an accessible transition pathway between them. The identification of this novel binding trench expands our understanding of ERRα′s ligand binding domain, offering new avenues for small-molecule drug discovery and selective modulation of ERRα activity.

## 1 Introduction

**FIGURE 1:**
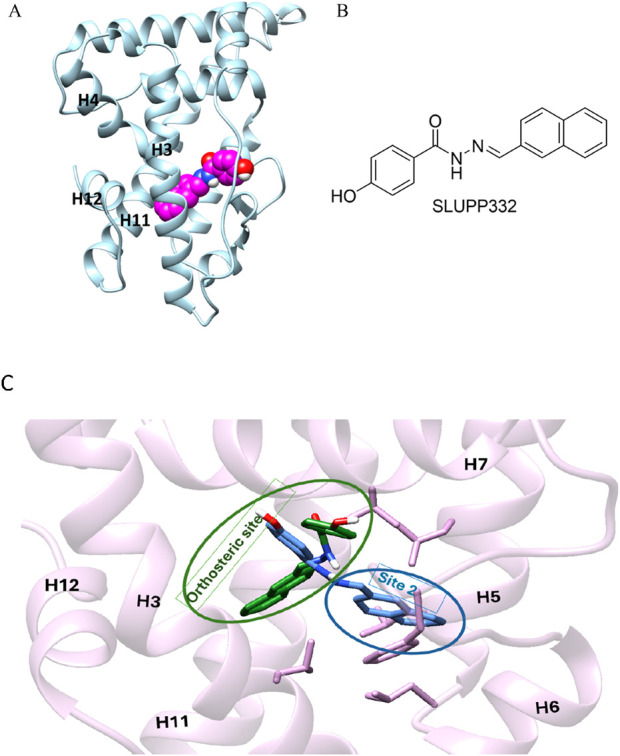
**(A)** Initial structure of the ligand binding domain ERRα shown in blue; helices 3,4, 11, and 12 are labeled in black; and the ligand, SLUPP332, is modeled in the ligand binding pocket (LBP) shown in the sphere (pink carbons). **(B)** Chemical structure of SLUPP332. **(C)** Overlay of two distinct ligand-binding orientations identified in MD simulations of ERRα. The receptor’s secondary structure and side chains are depicted in pink. The ligand orientation within the orthosteric site is shown in green, while the orientation in the novel binding trench (Site 2, S2) is shown in blue.

Estrogen-related receptor α (ERRα) is an orphan nuclear hormone receptor that regulates gene expressions related to anti-inflammatory activities, oxidative phosphorylation, biogenesis, and fatty acid metabolism (Audet-Walsh and Giguére, 2015; Huss et al., 2015; Mootha et al., 2003; Ranhotra, 2015). Recent studies reported the promising therapeutic importance of ERRα in the treatment of heart failure, kidney diseases, and metabolic disorders (Xu et al., 2024; Wang et al., 2023; Billon et al., 2023). The ligand binding domain (LBD) comprises 12 helices that harbor a hydrophobic ligand-binding pocket referred to as the orthosteric site (Figure 1A) (Greschik et al., 2002; Kallen et al., 2007). Despite numerous attempts to develop synthetic agonists for ERRα, the current ligands exhibit inadequate pharmacokinetic characteristics and a lack of selectivity (Shinozuka et al., 2021; Kallen et al., 2007; Shahien et al., 2020). These limitations impede research efforts aimed at unraveling the pharmacological behavior of this receptor. A greater understanding of the mechanistic events associated with ERRα binding is critical for the design of novel and selective agonists of this receptor. The current understanding of ligand binding implies that ligands exhibit a stronger binding to specific conformations within the dynamic ensemble of their protein targets. This process of binding, known as conformational selection, drives the selection of higher affinity conformers, forming energetically more stable complexes that dissociate in the presence of substantial conformational changes (Miller and Dill, 1997; Du et al., 2016; Seo et al., 2014; Boehr et al., 2009; Wei et al., 2016). There is a prevalent belief supported by a diverse range of structural data that ligands bind in a singular orientation in the target protein (Popovych et al., 2006; Boehr et al., 2006; Vogt and Di Cera, 2013; Onuchic et al., 1997). In contrast, a recent set of studies reported several targets where ligands could bind in several orientations instead of only one singular orientation in a notion referred to as dynamic ligand binding (Bruning et al., 2010; Bock et al., 2014; Hughes et al., 2012). The phenomenon of dynamic ligand binding was initially discovered in estrogen receptors, and it was later reported for the muscarinic M_2_ receptor and peroxisome proliferator-activated receptor gamma (PPARγ) (Bock et al., 2014; Bruning et al., 2010; Hughes et al., 2012). Further studies reported that ligand-binding dynamics directs unique pharmacological and signaling pathways (Bock et al., 2014; Srinivasan et al., 2013).

As part of our ongoing efforts to elucidate the molecular basis of ligand recognition and binding in nuclear hormone receptors (Kchouk and Hegazy, 2022; Griffett et al., 2020; Elgendy et al., 2022; Shahien et al., 2020; Du et al., 2017; Yu et al., 2017; Lou et al., 2014; Murray et al., 2022; Griffett et al., 2020), we carried out molecular dynamics simulations on ERRα in complex with the agonist SLUPP332 (Billon et al., 2023). SLUPP332 is a synthetic pan-agonist for estrogen-related receptors, recognized for its ability to mimic the effects of physical exercise, and is referred to as an “exercise mimetic.” Additionally, it has been shown to improve mitochondrial function in conditions such as heart failure and aging-related kidney dysfunction (Wang et al., 2023; Xu et al., 2024). These simulations revealed a dynamic interconversion of the ligand between two distinct binding orientations on a nanosecond timescale, a phenomenon that can be referred to as dynamic ligand binding (Figure 1C). Interestingly, one of these binding modes uncovers a novel binding trench within the ERRα Ligand binding domain (LBD) (Figure 1C), presenting new opportunities for small-molecule drug discovery.

## 2 Materials and methods

A set of three independent molecular dynamics trajectories of ERRα bound with SLUPP332 were modeled. Each simulation ran for 1,000 ns, with a total sampling time of 3,000 ns. The stability of the simulations was evaluated using the root mean square deviation (RMSD) and root mean square fluctuation (RMSF) of protein backbone atoms, as well as the RMSD of the ligand (Supplementary Figures S1–S3). The initial coordinates of the ERRα were taken from the apo ERRα crystal structure (PDB:1XB7) (Kallen et al., 2004). The ligand SLUPP332 was constructed by superimposing the protein backbone with the protein backbone of the ERRγ-GSK4716 complex (PDB:2GPP) (Wang L. et al., 2006; Pettersen et al., 2004). SLUPP332 was modeled by modifying the structurally similar GSK4716 compound using Maestro (Schrödinger, 2021). Molecular dynamics (MD) simulations were performed with the AMBER18 software package (Case et al., 2018). Ligand parameters were assigned according to the general AMBER force field (GAFF) and the corresponding AM1BCC charges using Antechamber (Wang et al., 2004; Wang J. et al., 2006). The FF14SB forcefield parameters were used for all receptor residues (Maier et al., 2015). The Tleap module was used to neutralize and solvate the complexes using an octahedral water box of TIP3P water molecules (Jorgensen et al., 1983).

The system was first energy-minimized using the steepest descent and conjugate gradient methods. After minimization, the system is gradually heated to 300 K over 100 Ps while keeping weak restraints on the solute and the ligand. The system was then equilibrated in the isothermal−isobaric ensemble (NPT) for 100 ps with restraints on the ligand. Three MD trajectories were propagated using the NVT ensemble with no restraints for 100 ns each using the GPU-accelerated version of the PMEMD program. All production simulations were performed at 1 atm and 300 K, maintained with the Berendsen barostat and thermostat, respectively. The periodic boundary conditions and the particle mesh Ewald method (grid spacing of 1 Å) were used for treating long-range electrostatic interactions with a uniform neutralizing plasma. The SHAKE algorithm was used to keep bonds involving H atoms at their equilibrium length, allowing the use of a 2fs time step for the integration of Newton’s equations. The 2D free energy map and the per residue RMSD of protein and ligand atoms and amino acid residues Phe328 and Phe382 were calculated using the CPPTRAJ module (Roe and Cheatham, 2013). Pictures were generated using UCSF Chimera and Maestro (Pettersen et al., 2004; Schrödinger, 2019). All plots were performed using Gnuplot, version 5.4 (http://gnuplot.info).

## 3 Results

**FIGURE 2:**
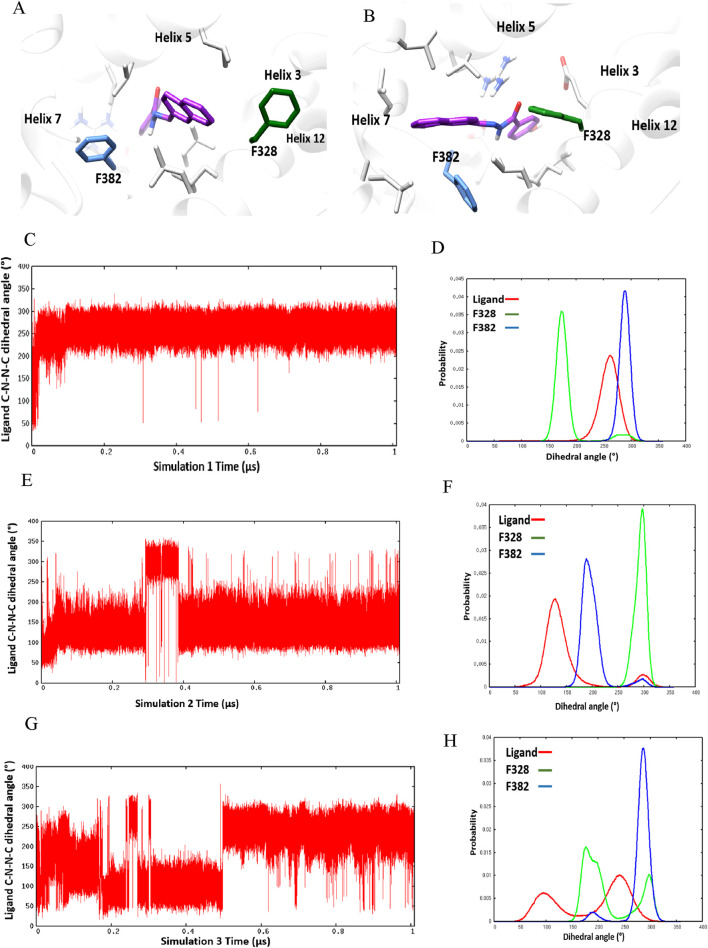
Ligand-binding orientation in **(A)** the orthosteric site (S1) and **(B)** the novel binding trench (S2). **(C–H)** Time course behavior of the ligand’s C-N-N-C dihedral angle rotation and corresponding histogram plots of the ligand’s C-N-N-C and *x*_1_ angles of Phe328 and Phe382 in Simulation 1 **(C, D)**, Simulation 2 **(E, F)**, and Simulation 3 **(G, H)**.

**FIGURE 3:**
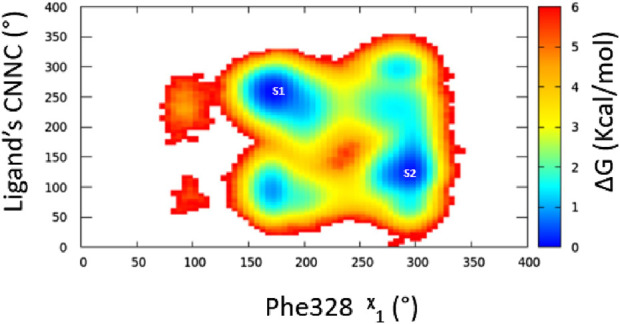
Free energy surface for ligand dynamic orientation in the LBP of ERRα. The points labeled S1 and S2 represent ligand orientation in the orthosteric site and Site 2, respectively.

Three separate molecular dynamics simulations (one microsecond each) were performed on ERRα bound with the pan-agonist SLUPP332 (Figure 1B). Simulations revealed dynamic ligand binding of SLUPP332, where the ligand’s naphthalene group flipped its initial orientation spontaneously (Figure 2A) into a novel binding trench that will be referred to as Site 2 (S2) (Figure 2B). Ligand orientation was monitored by measuring the dihedral angle rotation around the ligand’s C-N-N-C dihedral angle (Figure 2). In Simulation 1, the ligand maintained its orientation mainly in the orthosteric site with the C-N-N-C dihedral angle holding a value of ∼250° (Figure 2C). Conversely, in Simulation 2, the ligand’s naphthalene group transitioned to Site 2, maintaining a predominantly dihedral angle of ∼120° throughout most of the simulation. The third simulation exhibited spontaneous rotation of the ligand’s naphthalene group between both orientations (Figure 2G). The change of the ligand’s orientation is correlated with the conformational change of either Phe328 or Phe382 (Figure 2). In Simulation 1, where the ligand is predominantly stable in the orthosteric site, the Phe328 side chain flipped away from the orthosteric site, closer to helix 12 (Figure 2A), and the Phe328 *x*_1_ angle occupied predominantly a value of 175° while the Phe382 *x*_1_ angle occupied predominantly a value of 290°. The flexibility of the Phe328 side chain was observed previously in the X-ray structure of ERRα-bound with the inverse agonist cyclohexyl methyl amine, where the side chain of Phe328 changed its conformation to accommodate the inverse agonist binding (PDB: 2PJL) (Kallen et al., 2007). The same amino acid residue Phe328 on helix 3 in the ligand-binding pocket was also reported to be essential for the constitutive activity of ERRα and its mutation to alanine, leading to the loss of the ERRα constitutive activity (Chen et al., 2001). In Simulation 2, the ligand’s naphthalene group flipped almost 180° from the initial binding mode into a novel binding trench, S2 (Figure 2B). In accordance with the ligand’s orientation change, Phe382 was observed to move downward, providing the necessary space for the ligand’s naphthalene group to bind in this orientation (Figure 2B and Supplementary Video 1). The Phe382 *x*_1_ angle had a value of 200° while the Phe328 *x*_1_ angle had a value of 125° (Figure 2F). In Simulation 3, the ligand’s naphthalene group underwent a spontaneous orientation shift between both orientations. The side chain of Phe382 predominantly adopted a conformation resembling that in Simulation 1, with an *x*_1_ angle primarily approximately 290°, indicating a predominantly open pocket at Site 2. Meanwhile, Phe328 alternated between two conformations similar to those observed in Simulations 1 and 2 (Supplementary Video 2). From our simulation results, a 2D relative free energy map was generated by analyzing the combined trajectory of all three simulations. This map is based on the rotation of the ligand’s C-N-N-C dihedral angle and the *x*_1_ angle of Phe328 (Figure 3). It reveals the presence of two distinct low-energy populations of two ligand-bound orientations stabilized by one dynamic ligand. Both ligands’ bound states have comparable ∆G values, and the transition between them is facile, with an activation barrier of no more than 3.5 kcal mol^−1^. The variation in ligand-binding orientation between the orthosteric site (S1) and the newly identified trench (S2) correlates with the rotation of the Phe328 *x*_1_ angle (Figure 3). Specifically, the orientation of the ligand within the novel binding trench, S2, is energetically preferable when the Phe328 *x*_1_ angle is approximately 300°, Whereas ligand orientation is favorable in the orthosteric site (S1) when the Phe328 *x*_1_ angle is around 170°.

**FIGURE 4:**
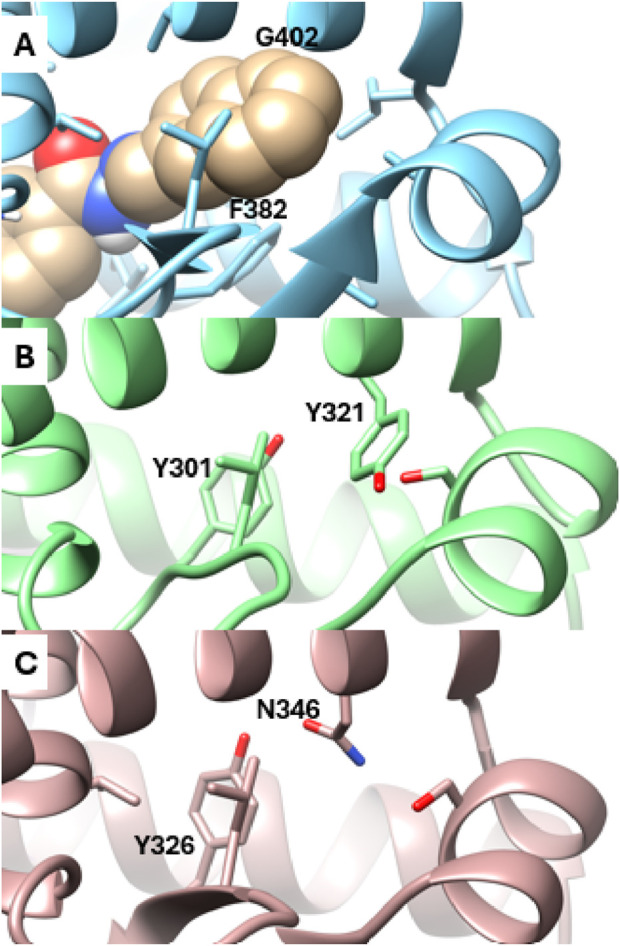
Detailed view of the Site 2 region. **(A)** Snapshot from MD simulations of ERRα bound with SLUPP332 (shown in sphere representation). The corresponding region is depicted in the X-ray structures of ERRβ **(B)** and ERRγ **(C)**.

Analysis of the X-ray structures of the other ERR isoforms, ERRβ and ERRγ, indicates that the newly discovered site is unique to ERRα. In ERRα, this site is gated by two amino acid residues, Phe382 and Gly402, which correspond to Tyr301 and Tyr321 in ERRβ and Tyr326 and Asn346 in ERRγ, respectively (Figure 4). The presence of Gly402 in ERRα, instead of Tyr321 and Asn346 in ERRβ and ERRγ, creates a vacant space in ERRα that becomes more favorable for ligand binding as the *x*_1_ angle of Phe382 predominantly adopts a value of 290° (Figures 1B, D).

## 4 Discussion

Through classical molecular dynamics simulations, we show that the ERRα agonist, SLUPP332, dynamically switches between two distinct binding orientations. Furthermore, the simulations unveiled a previously uncharacterized trench adjacent to the orthosteric site that opens because of conformational changes of the ligand, Phe328, and Phe382. This dynamic behavior of ligand binding was previously observed using hydrogen–deuterium exchange and solution NMR experiments in other nuclear receptors (PPARγ and ERα) as well as in a GPCR receptor, indicating a novel mechanism of allosteric signaling (Bock et al., 2014; Hughes et al., 2012; Bruning et al., 2010). Further experimental data indicate that the orientation of the ligand influences receptor-graded activity and cellular response, presenting new opportunities for designing drugs with targeted pharmacological effects (Bock et al., 2014; Bruning et al., 2010). These insights provide a detailed understanding of the molecular mechanisms underlying the agonist ligand binding SLUPP332 to ERRα, facilitating innovative strategies for designing modulators that specifically target ligand dynamics and flexibility. Notably, this unique feature of ERRα underscores the importance of molecular simulations in elucidating crucial insights into ligand-binding dynamics. Molecular simulations have proven pivotal in the characterization of various conformational states of proteins, particularly in essential drug discovery initiatives, such as in the cases of HIV-1 protease and urease (Hornak et al., 2006; Roberts et al., 2012).

## Data Availability

The original contributions presented in the study are included in the article/Supplementary Material, further inquiries can be directed to the corresponding author.

## References

[B1] Audet-Walsh É. Giguére V. (2015). The multiple universes of estrogen-related receptor α and γ in metabolic control and related diseases. Acta Pharmacol. Sin. 36 (1), 51–61. 10.1038/aps.2014.121 25500872 PMC4571319

[B2] Billon C. Sitaula S. Banerjee S. Welch R. Elgendy B. Hegazy L. (2023). Synthetic errα/β/γ agonist induces an ERRα-dependent acute aerobic exercise response and enhances exercise capacity. ACS Chem. Biol. 18 (4), 756–771. 10.1021/acschembio.2c00720 36988910 PMC11584170

[B3] Bock A. Chirinda B. Krebs F. Messerer R. Bätz J. Muth M. (2014). Dynamic ligand binding dictates partial agonism at a G protein–coupled receptor. Nat. Chem. Biol. 10 (1), 18–20. 10.1038/nchembio.1384 24212135

[B4] Boehr D. D. Dyson H. J. Wright P. E. (2006). An NMR perspective on enzyme dynamics. Chem. Rev. 106 (8), 3055–3079. 10.1021/cr050312q 16895318

[B5] Boehr D. D. Nussinov R. Wright P. E. (2009). The role of dynamic conformational ensembles in biomolecular recognition. Nat. Chem. Biol. 5 (11), 789–796. 10.1038/nchembio.232 19841628 PMC2916928

[B6] Bruning J. B. Parent A. A. Gil G. Zhao M. Nowak J. Pace M. C. (2010). Coupling of receptor conformation and ligand orientation determine graded activity. Nat. Chem. Biol. 6 (11), 837–843. 10.1038/nchembio.451 20924370 PMC2974172

[B7] Case D. A. Ben-Shalom I. Y. Brozell S. R. Cerutti D. S. Cheatham T. E. III Cruzeiro V. W. D. (2018). Amber 2018. San Francisco: University of California.

[B8] Chen S. Zhou D. Yang C. Sherman M. (2001). Molecular basis for the constitutive activity of estrogen-related receptor alpha-1. J. Biol. Chem. 276 (30), 28465–28470. 10.1074/jbc.M102638200 11356845

[B9] Du X. Li Y. Xia Y.-L. Ai S.-M. Liang J. Sang P. (2016). Insights into protein–ligand interactions: mechanisms, models, and methods. Int. J. Mol. Sci. 17, 144. 10.3390/ijms17020144 26821017 PMC4783878

[B10] Du Y. Song L. Zhang L. Ling H. Zhang Y. Chen H. (2017). The discovery of novel, potent ERR-alpha inverse agonists for the treatment of triple negative breast cancer. Eur. J. Med. Chem. 136, 457–467. 10.1016/j.ejmech.2017.04.050 28525844

[B11] Elgendy B. Griffett K. Hegazy L. Di Fruscia P. Sample K. Schoepke E. (2022). Synthesis and structure activity relationship of the first class of LXR inverse agonists. Bioorg Chem. 119, 105540. 10.1016/j.bioorg.2021.105540 34902646

[B12] Greschik H. Wurtz J.-M. Sanglier S. Bourguet W. van Dorsselaer A. Moras D. (2002). Structural and functional evidence for ligand-independent transcriptional activation by the estrogen-related receptor 3. Mol. Cell 9 (2), 303–313. 10.1016/s1097-2765(02)00444-6 11864604

[B13] Griffett K. Bedia-Diaz G. Hegazy L. de Vera I. M. S. Wanninayake U. S. Billon C. (2020). The orphan nuclear receptor TLX is a receptor for synthetic and natural retinoids. Cell Chem. Biol. 27 (10), 1272–1284.e4. 10.1016/j.chembiol.2020.07.013 32763139

[B14] Hornak V. Okur A. Rizzo R. C. Simmerling C. (2006). HIV-1 protease flaps spontaneously close to the correct structure in simulations following manual placement of an inhibitor into the open state. J. Am. Chem. Soc. 128 (9), 2812–2813. 10.1021/ja058211x 16506755 PMC2555982

[B15] Hughes T. S. Chalmers M. J. Novick S. Kuruvilla D. S. Chang M. R. Kamenecka T. M. (2012). Ligand and receptor dynamics contribute to the mechanism of graded PPARγ agonism. Structure 20 (1), 139–150. 10.1016/j.str.2011.10.018 22244763 PMC3278220

[B16] Huss J. M. Garbacz W. G. Xie W. (2015). Constitutive activities of estrogen-related receptors: transcriptional regulation of metabolism by the ERR pathways in health and disease. Biochimica Biophysica Acta 1852, 1912–1927. 10.1016/j.bbadis.2015.06.016 26115970

[B17] Jorgensen W. L. Chandrasekhar J. Madura J. D. Impey R. W. Klein M. L. (1983). Comparison of simple potential functions for simulating liquid water. J. Chem. Phys. 79 (2), 926–935. 10.1063/1.445869

[B18] Kallen J. Lattmann R. Beerli R. Blechschmidt A. Blommers M. J. J. Geiser M. (2007). Crystal structure of human estrogen-related receptor alpha in complex with a synthetic inverse agonist reveals its novel molecular mechanism. J. Biol. Chem. 282 (32), 23231–23239. 10.1074/jbc.M703337200 17556356

[B19] Kallen J. Schlaeppi J. M. Bitsch F. Filipuzzi I. Schilb A. Riou V. (2004). Evidence for ligand-independent transcriptional activation of the human estrogen-related receptor alpha (ERRalpha): crystal structure of ERRalpha ligand binding domain in complex with peroxisome proliferator-activated receptor coactivator-1alpha. J. Biol. Chem. 279, 49330–49337. 10.1074/jbc.M407999200 15337744

[B20] Kchouk S. Hegazy L. (2022). Pharmacophore modeling for biological targets with high flexibility: LXRβ case study. Med. Drug Discov. 15, 100135. 10.1016/j.medidd.2022.100135

[B21] Lou X. Toresson G. Benod C. Suh J. H. Philips K. J. Webb P. (2014). Structure of the retinoid X receptor α-liver X receptor β (RXRα-LXRβ) heterodimer on DNA. Nat. Struct. Mol. Biol. 21 (3), 277–281. 10.1038/nsmb.2778 24561505

[B22] Maier J. A. Martinez C. Kasavajhala K. Wickstrom L. Hauser K. E. Simmerling C. (2015). Ff14SB: improving the accuracy of protein side chain and backbone parameters from Ff99SB. J. Chem. Theory Comput. 11 (8), 3696–3713. 10.1021/acs.jctc.5b00255 26574453 PMC4821407

[B23] Miller D. W. Dill K. A. (1997). Ligand binding to proteins: the binding landscape model. Protein Sci. 6 (10), 2166–2179. 10.1002/pro.5560061011 9336839 PMC2143563

[B24] Mootha V. K. Bunkenborg J. Olsen J. V. Hjerrild M. Wisniewski J. R. Stahl E. (2003). Integrated Analysis of protein composition, tissue diversity, and gene regulation in mouse mitochondria. Cell 115, 629–640. 10.1016/S0092-8674(03)00926-7 14651853

[B25] Murray M. H. Valfort A. C. Koelblen T. Ronin C. Ciesielski F. Chatterjee A. (2022). Structural basis of synthetic agonist activation of the nuclear receptor REV-ERB. Nat. Commun. 13 (1), 7131. 10.1038/s41467-022-34892-4 36414641 PMC9681850

[B26] Onuchic J. N. Luthey-Schulten Z. Wolynes P. G. (1997). Theory of protein folding: the energy landscape perspective. Annu. Rev. Phys. Chem. 48, 545–600. 10.1146/annurev.physchem.48.1.545 9348663

[B27] Pettersen E. F. Goddard T. D. Huang C. C. Couch G. S. Greenblatt D. M. Meng E. C. (2004). UCSF chimera—a visualization system for exploratory research and Analysis. J. Comput. Chem. 25 (13), 1605–1612. 10.1002/jcc.20084 15264254

[B28] Popovych N. Sun S. Ebright R. H. Kalodimos C. G. (2006). Dynamically driven protein allostery. Nat. Struct. Mol. Biol. 13 (9), 831–838. 10.1038/nsmb1132 16906160 PMC2757644

[B29] Ranhotra H. S. (2015). Estrogen-related receptor alpha and mitochondria: tale of the titans. J. Recept. Signal Transduct. 35, 386–390. 10.3109/10799893.2014.959592 25222219

[B30] Roberts B. P. Miller B. R. Roitberg A. E. Merz K. M. Jr. (2012). Wide-open flaps are key to urease activity. J. Am. Chem. Soc. 134 (24), 9934–9937. 10.1021/ja3043239 22670767 PMC3384008

[B31] Roe D. R. Cheatham T. E. (2013). PTRAJ and CPPTRAJ: software for processing and Analysis of molecular dynamics trajectory data. J. Chem. Theory Comput. 9 (7), 3084–3095. 10.1021/ct400341p 26583988

[B32] Schrödinger (2019). Schrödinger release 2019-1: Maestro. New York, NY: Schrödinger, LLC.

[B33] Schrödinger (2021). Schrödinger release 2021-3: MacroModel. New York, NY: Schrödinger, LLC.

[B34] Seo M.-H. Park J. Kim E. Hohng S. Kim H.-S. (2014). Protein conformational dynamics dictate the binding affinity for a ligand. Nat. Commun. 5 (1), 3724. 10.1038/ncomms4724 24758940

[B35] Shahien M. Elagawany M. Sitaula S. Goher S. S. Burris S. L. Sanders R. (2020). Modulation of estrogen-related receptors subtype selectivity: conversion of an ERRβ/γ selective agonist to ERRα/β/γ pan agonists. Bioorg Chem. 102, 104079. 10.1016/j.bioorg.2020.104079 32683181 PMC9137328

[B36] Shinozuka T. Ito S. Kimura T. Izumi M. Wakabayashi K. (2021). Discovery of a novel class of ERRα agonists. ACS Med. Chem. Lett. 12 (5), 817–821. 10.1021/acsmedchemlett.1c00100 34055231 PMC8155236

[B37] Srinivasan S. Nwachukwu J. C. Parent A. A. Cavett V. Nowak J. Hughes T. S. (2013). Ligand-binding dynamics rewire cellular signaling via estrogen receptor-α. Nat. Chem. Biol. 9 (5), 326–332. 10.1038/nchembio.1214 23524984 PMC3631275

[B38] Vogt A. D. Di Cera E. (2013). Conformational selection is a dominant mechanism of ligand binding. Biochemistry 52 (34), 5723–5729. 10.1021/bi400929b 23947609 PMC3791601

[B39] Wang J. Wang W. Kollman P. A. Case D. A. (2006b). Automatic atom type and bond type perception in molecular mechanical calculations. J. Mol. Graph Model 25 (2), 247–260. 10.1016/j.jmgm.2005.12.005 16458552

[B40] Wang J. Wolf R. M. Caldwell J. W. Kollman P. A. Case D. A. (2004). Development and testing of a general amber force field. J. Comput. Chem. 25 (9), 1157–1174. 10.1002/jcc.20035 15116359

[B41] Wang L. Zuercher W. J. Consler T. G. Lambert M. H. Miller A. B. Orband-Miller L. A. (2006a). X-ray crystal structures of the estrogen-related receptor-γ ligand binding domain in three functional states reveal the molecular basis of small molecule regulation. J. Biol. Chem. 281, 37773–37781. 10.1074/jbc.M608410200 16990259

[B42] Wang X. X. Myakala K. Libby A. E. Krawczyk E. Panov J. Jones B. A. (2023). Estrogen-related receptor agonism reverses mitochondrial dysfunction and inflammation in the aging kidney. Am. J. Pathol. 193 (12), 1969–1987. 10.1016/j.ajpath.2023.07.008 37717940 PMC10734281

[B43] Wei G. Xi W. Nussinov R. Ma B. (2016). Protein ensembles: how does nature harness thermodynamic fluctuations for life? The diverse functional roles of conformational ensembles in the cell. Chem. Rev. 116 (11), 6516–6551. 10.1021/acs.chemrev.5b00562 26807783 PMC6407618

[B44] Xu W. Billon C. Li H. Wilderman A. Qi L. Graves A. (2024). Novel pan-ERR agonists ameliorate heart failure through enhancing cardiac fatty acid metabolism and mitochondrial function. Circulation 149 (3), 227–250. 10.1161/CIRCULATIONAHA.123.066542 37961903 PMC10842599

[B45] Yu D. D. Huss J. M. Li H. Forman B. M. (2017). Identification of novel inverse agonists of estrogen-related receptors ERRγ and ERRβ. Bioorg Med. Chem. 25 (5), 1585–1599. 10.1016/j.bmc.2017.01.019 28189393

