# Supplementary material for: Uncovering a novel binding trench in ERRα: insights from molecular simulations
Source: Front Mol Biosci. 2025 Feb 27;12:1523932. doi: 10.3389/fmolb.2025.1523932 (PMC11903277; doi:10.3389/fmolb.2025.1523932)
Supplement: Supplementary file 1 [file Supplementaryfile1.docx]

Uncovering a Novel Binding Trench in ERRα: Insights from Molecular Simulations

Lamees Hegazy^1,2^

^1^Center for Clinical Pharmacology, Washington University School of Medicine in Saint Louis and University of Health Sciences and Pharmacy in Saint Louis, St. Louis, MO 63110, USA.

^2^Department of Pharmaceutical and Administrative Sciences, Saint Louis College of Pharmacy, University of Health Sciences & Pharmacy in St. Louis, St. Louis, MO 63110, USA.


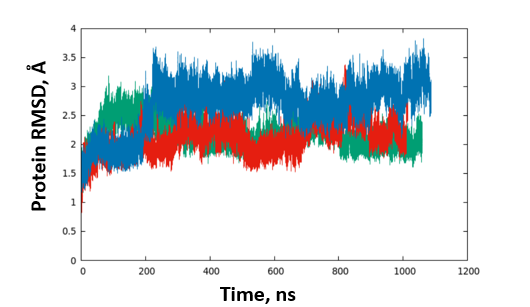


**Supplementary Figure 1.** RMSD of protein backbone atoms across three simulations. The RMSD plot is displayed in green for Simulation 1, red for Simulation 2, and blue for Simulation 3.


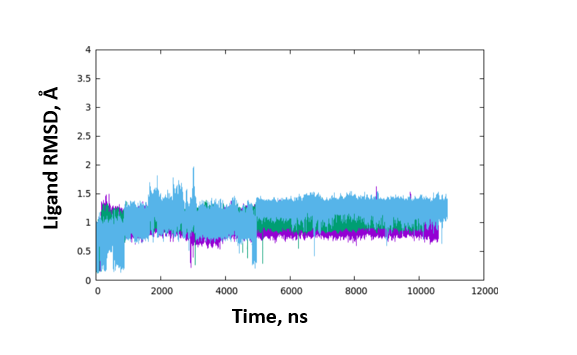


**Supplementary Figure 2.** RMSD of ligand’s heavy atoms across three simulations. The RMSD plot is displayed in purple for Simulation 1, green for Simulation 2, and blue for Simulation 3.


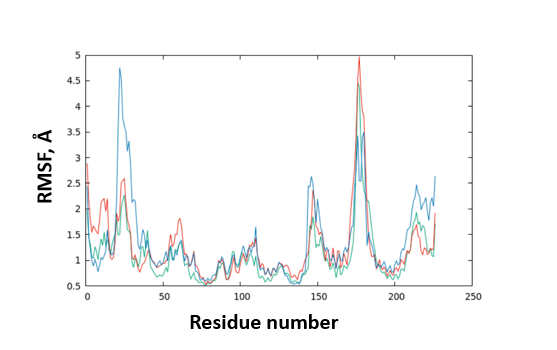


**Supplementary Figure 3.** RMSF of protein backbone atoms across three simulations. The RMSF plot is displayed in green for Simulation 1, red for Simulation 2, and blue for Simulation 3. Receptor regions with RMSF > 3 Å, correspond to flexible loop regions, loop 1 (amino acid residues: 12 -27) and loop 2 (amino acid residues: 172 - 191).

Supplementary video 1. Movie of simulation 2 showing change of ligand binding orientation from initial position, orthosteric site (S1) into the novel binding trench, S2. The initial model of ERRα bound with SLUPP332 is shown in light grey. The same model after running molecular dynamics simulations is shown in blue. Helix 12 is shown in red.

Supplementary video 2. Movie of the combined simulations trajectory showing interconversion of ligand binding orientation between the orthosteric site and the novel binding trench, S2. Receptor colored in light blue, helix12 in grey, ligand carbon atoms are colored in pink, Phe328 is colored in red and Phe382 is colored in purple.
